# From proteome to pathogenesis: investigating polycystic ovary syndrome with Mendelian randomization analysis

**DOI:** 10.3389/fendo.2024.1442483

**Published:** 2024-09-09

**Authors:** Jiaqi Zhang, Yuqing Li, Aixia Gong, Jingmin Wang

**Affiliations:** ^1^ Department of Digestive Endoscopy, The First Affiliated Hospital of Dalian Medical University, Dalian, Liaoning, China; ^2^ Department of Obstetrics and Gynecology, The First Affiliated Hospital of Dalian Medical University, Dalian, Liaoning, China

**Keywords:** polycystic ovary syndrome, metabolism, proteome, Mendelian randomization, bioinformatics

## Abstract

**Background:**

Polycystic ovary syndrome (PCOS) is defined by oligo/anovulation, hyperandrogenism, and polycystic ovaries with uncertain pathogenesis. The proteome represents a substantial source of therapeutic targets, and their coding genes may elucidate the mechanisms underlying PCOS. However, reports on the profiles of the human plasma protein-coding genes and PCOS are limited. Here, we aimed to investigate novel biomarkers or drug targets for PCOS by integrating genetics and the human plasma proteome.

**Methods:**

Our study acquired the protein quantitative trait loci from DECODE Genetics, offering 4,907 proteins in 35,559 individuals while obtaining PCOS summary statistics by accessing the FinnGen biobank (1,639 cases and 218,970 controls) and the genome-wide association study catalog (797 cases and 140,558 controls). Herein, we sequentially used two-sample Mendelian randomization (MR) analyses and colocalization to verify the causal link between candidate proteins, their coding genes, and PCOS. Further PCOS data download was conducted by accessing the Gene Expression Omnibus and Zenodo platforms. Gene expression level analysis, pathway enrichment analysis, immune cell infiltration, and transcription factor prediction were performed, aiming at detecting specific cell types with enriched expression and exploring potential optimized treatments for PCOS.

**Results:**

MR analysis revealed 243 protein-coding genes with a causal relationship to PCOS risk, of which 12 were prioritized with the most significant evidence. Through colocalization analysis, three key genes, CUB domain-containing protein 1 (*CDCP1*), glutaredoxin 2 (*GLRX2*), and kirre-like nephrin family adhesion molecule 2 (*KIRREL2*), were identified. Subsequently, the three genes were strongly related to immune function and metabolism in terms of biological significance. In single-cell analysis, the expression levels of genes in ovarian theca cells were explored.

**Conclusion:**

Overall, three protein-coding genes (*CDCP1*, *GLRX2*, and *KIRREL2*) may be related to a higher PCOS risk, suggesting that they may be entry points for exploration of PCOS pathogenesis and treatment, warranting further clinical investigations.

## Introduction

Polycystic ovary syndrome (PCOS) constitutes a highly heterogeneous reproductive endocrine disease affecting women of reproductive age that is defined by a wide range of symptoms and can be diagnosed using several criteria, resulting in a prevalence of 4–21% (1–5). PCOS diagnosis typically demands the inclusion of at least two out of these three symptoms: oligovulation-anovulation, hyperandrogenism (clinical symptoms and/or androgen excess), and polycystic ovarian morphology (2). Additionally, patients with PCOS are often associated with substantial metabolic abnormalities, including diabetes, insulin resistance (IR), obesity, adipose tissue dysfunction, and reproductive disorders (6, 7). Considering the high heterogeneity and clinical variability of PCOS, besides the interplay of genetic, endocrine, and environmental factors that may be implicated in PCOS development, its specific pathogenesis has not been fully elucidated (8). It is indicated that increased production of ovarian and/or adrenal androgen, partial folliculogenesis arrest, IR, and neuroendocrine axis dysfunction seem to contribute to PCOS to different degrees (9). Currently, as PCOS has no effective cure (10), lifestyle modifications (involving exercise, diet, weight, mood, and sleep) are recommended as the first-line therapy for PCOS (11, 12), and treatment focuses primarily on managing symptoms in clinical settings (13).

PCOS is a complex polygenic syndrome characterized by genetic alterations that underlie a wide range of pathophysiological processes, including metabolism, endocrinology, immunity, and inflammation (14, 15). Although the contribution of genetic factors is estimated to be less than 10%, they play an important role in the clinical manifestations of the disease (16). Through examining a large twin cohort of 1332 monozygotic and 1873 dizygotic twin sisters,a study concluded that the genetic component contributes to over 70% of PCOS pathogenesis (17). A multitude of candidate genes has been discovered,including genes involved in insulin action, androgen biosynthesis, and gonadal function (18). Distinct PCOS subtypes are associated with different genetic heterogeneity, therefore, the establishment of candidate genes is still in the exploratory stage. Exploring protein-coding genes may play an essential role in proposing PCOS biomarkers and drug targets. Recently, several methods have been used to identify potential biomarkers. For instance, it has been discovered that the elevated miR-26b expression, a type of extracellular vesicle, may promote the impaired hormone levels of PCOS (19), which consequently makes it a possible biomarker for PCOS. Additionally, through the microbiomes inquiry, it is suggested that fecal microbiota transplantation may constitute a novel therapeutic strategy for the management of PCOS (20). However, reports on the profiles of the human plasma protein-coding genes and PCOS are scarce. Therefore, the study directly investigates the relationship between these plasma protein-coding genes and PCOS and hypothesizes that the regulatory mechanisms of these genes may serve as a basis for developing future drug targets/novel biomarkers/tailor treatment for PCOS.

Proteins, most circulating in the blood, originating from cellular leakage or active secretion, act as key regulators of molecular pathways (21). They include immune-related proteins (such as immunoglobulins and cytokines), which play a critical role in mediating these processes. Consequently, proteomic research has substantially enhanced our understanding of these mechanisms, contributing to the identification of potential biomarkers, therapeutic targets, and insights into disease pathogenesis (22). Notably, extensive proteomic investigations have discovered over 18,000 protein quantitative trait loci (pQTL) that encompass over 4,800 proteins, providing excellent data resources for thoroughly understanding the causal influence of proteins in circulation (23). Mendelian randomization (MR) is designed to detect causal effects impartially through genetic variations (GVs) as instrumental variables (IVs) to associate exposure with outcomes (24, 25). The inherent random distribution and stability of these GVs allow differences in exposure to explain the outcome disparities between individuals with and without GVs (26). Consequently, MR is not subject to the typical confounding factors and reverse causation issues prevalent in epidemiology (26). Integrating genome-wide association study (GWAS) findings of women with PCOS with pQTL data may provide probable explanations (potential causal genes and drug targets) for immune dysregulation in PCOS (27). Additionally, single-cell RNA sequencing (scRNA-seq) analysis allows more in-depth exploration of diseases in the cellular and molecular dimensions, revealing how genetic factors interact with cellular malfunctions and molecular changes. Given the limited research on the impact of pQTLs in PCOS, conducting thorough and systematic studies is imperative to evaluate the causal influences of circulating proteins and their coding genes on PCOS.

Noteworthy, it is believed that PCOS is closely related to the imbalance of immune homeostasis. Immune cells, including macrophages and B/T cells, are vital in PCOS development by mediating the development of a chronic low-grade inflammatory state, closely related to PCOS clinical features that include obesity, IR, and hyperandrogenemia (28–31). In addition, other non-metabolic organismal pathological changes in PCOS (cardiac macrophage aggregation) are associated with immune cell activity (32). The imbalance of the immune microenvironment is expected to become a breakthrough point in treatment and medication for PCOS women. Treatments against potential immune targets may have unique advantages in restoring ovarian function, reducing IR, and increasing the chance of conception in PCOS patients. Only a few studies have specifically examined the subtle variations in the activity status among various immune cell types in patients with PCOS. Therefore, the correlation between immune cells and potential target genes needs to be further analyzed and discussed.

In summary, with the integration of genetics and the human plasma proteome, MR was used to explore the causal genes of PCOS. Due to the significance of the inflammatory immune mechanism behind PCOS onset and progression, pathway enrichment analysis has been performed to find whether potential pathways related to immunity and inflammation exist. Furthermore, the RNA-seq data of PCOS from ovaries were applied to investigate the association of immune cells and the three protein-coding genes. Meanwhile, scRNA-seq data have been collected to analyze the expression of the causal protein-coding genes. The aim of our study was to find novel biomarkers/drug targets for PCOS administration by integrating genetics and the human plasma proteome, thereby comprehending PCOS pathogenesis contributing to developing personalized treatment strategies and exploring potential biomarkers as well as drug targets for patients with PCOS.

## Materials and methods

### Data sources

The pQTL data were obtained via DECODE Genetics, which determined 4,907 proteins in 35,559 European ancestry individuals through the SomaScan assay v.4 (SomaLogic) (23). For outcome data sources, FinnGen is a database containing samples collected from Finnish biobanks and phenotyping data gathered from national health registers; 1,639 PCOS cases and 218, 970 controls were included in this study (Finngen _R10_E4_PCOS). Other summary outcome data were derived from the GWAS Catalog database (GCST90044902) and used for external validation. Currently, the GWAS Catalog is mapped to Genome Assembly and dbSNP Build, resulting in 797 cases and 140,558 controls in the PCOS validation set. **Table 1** lists the detailed data.

**Table 1:** Summary of data sources.

MR	Traits	Database	Number		SNPs	Population
Exposure	pQTL	deCODE	35,559		26,683	European
			Cases	Controls		
Outcome	PCOS	FinnGen biobank	1,639	218,970	19,674,513	European
	PCOS	GWAS Catalog	797	140,558	11,108,199	European
Bioinformatics			Cases	Controls	Data type	Population
	PCOS	GEO	7	3	Transcriptome	Asian
	PCOS	Zenodo	5	5	Single-cell	European

### IV selection

The selection of SNPs as IVs depended on the requirement to satisfy three assumptions of MR: a correlation with the exposure, independence from other confounding factors, and an effect on the result solely via the exposure (26). Our study established a threshold of P< 1e-6 for SNP selection. Additionally, we concurrently computed the F-value for the assessment of IV strength and selected IVs that exhibited a high correlation with F>10 (33). The threshold for the linkage disequilibrium coefficient was established as R^2^<0.001, using R^2^-values corresponding to a linkage disequilibrium distance of 10000 kb.

### MR analysis

To determine the connection between genetically predicted protein levels and PCOS, two-sample MR (TSMR) analyses were employed. Generally, the methods used to assess causality and determine the overall estimate of the influence of all cis and some cross-region gene expression on PCOS in whole blood included inverse-variance weighted (IVW), MR-Egger (MRE), weighted median (WME), and weighted modes (WMO) (34). Specifically, we employed the IVW method to calculate the MR effect estimates for proteins having over one instrument. The MRE method depended on the assumption that instrument strength is not influenced by direct effects. The WME method enables accurate estimation of causality in 50% of cases where the IVs are not valid. The WMO estimation method possesses a higher capability of detecting causal effects, a smaller bias, and a lower Type I error rate. The Wald ratio can be deployed as the sole statistical method to determine the SNP causal relationship. The study reported odds ratios (ORs) that indicate an increased risk of PCOS per standard deviation increase in plasma protein levels. We employed the MR leave-one-out sensitivity analysis to assess the influence of particular GVs on PCOS risk while conducting a heterogeneity test to evaluate genetic instrument heterogeneity using Q statistics. The insignificance of Q (*P*>0.05) indicated a lack of sufficient evidence to demonstrate heterogeneity presence among the effect sizes of the SNP, suggesting the statistical consistency of SNP impacts on disease risk.

### Co-localization analysis

Co-localization analyses were performed to estimate whether the connections between the identified proteins and PCOS were attributed to linkage disequilibrium using the R package Coloc (35). A posterior probability summary for each of the above five hypotheses was calculated using the packages (H0, H1, H2, H3, and H4). H0 represented no pQTL and no PCOS correlation; H1 and H2 indicated a link with pQTL but no PCOS link or vice versa; H3 reflected pQTL and GWAS connection but independent signals; and H4 referred to shared pQTL and PCOS correlation. In this study, colocalization support was defined as the posterior probability for a shared causal variant (PP.H4) > 0.65.

### Gene set variation analysis and gene set enrichment analysis

GSVA constitutes a nonparametric and unsupervised statistical technique utilized for the enrichment assessment of gene sets in transcriptome data. GSVA has the capability of converting gene-level changes into pathway-level changes through a comprehensive scoring of the gene set of interest and determining the sample biological function (36). GSEA is frequently employed to assess if a set of genes exhibits statistically significant variations between two biological phenotypes (37). Significantly enriched gene sets were identified and sorted based on their consistency scores (*P <*0.05).

### Immune cell infiltration analysis

ICI was estimated using the single-sample GSEA (ssGSEA) algorithm (38), which quantifies the relative infiltration of 29 immune cell types into the tumor immune microenvironment. Our study utilized ssGSEA for the data analysis of women with PCOS, aiming at inferring the connection between gene expression and immune-infiltrating cells. The R package “Hmisc” (https://CRAN.R-project.org/package=Hmisc) was used for correlation analysis and visualized by ggplot function. PCOS data download was conducted by accessing the Gene Expression Omnibus database (GSE34526) with seven cases and three controls (39).

### Transcription factor prediction

Herein, we predicted the TFs via the R package “RcisTarget,” conducting all calculations depending on motifs. The motif’s normalized enrichment score (NES) relies on the total motif number in the database. Besides the source data annotated motifs, we inferred additional annotation files on the basis of motif similarity and gene sequences. The NES for each motif was calculated via the area under the curve (AUC) distribution of all motifs in the gene set.

### Data processing of sc-RNA seq data of PCOS

The PCOS single-cell (SC) data file (theca cells from ovaries) downloaded from the Zenodo database, indicated in previous literature (40), included expression profile data from ten individuals, including five normal ovulatory women and five having PCOS. The processing of gene expression profiles was conducted through the Seurat package in R. Quality control, filtering, normalization, and subsequent analyses were performed. The positional relation between each cluster was acquired through T-distributed Stochastic Neighbor Embedding (tSNE) for nonlinear dimensionality reduction (41). Visualization of SC gene expression and co-expressed genes in PCOS were explored.

### Statistical analysis

R software (version 4.2.0) was utilized to conduct this study, setting *P*<0.05 as statistical significance.

## Results

**Figure 1:**
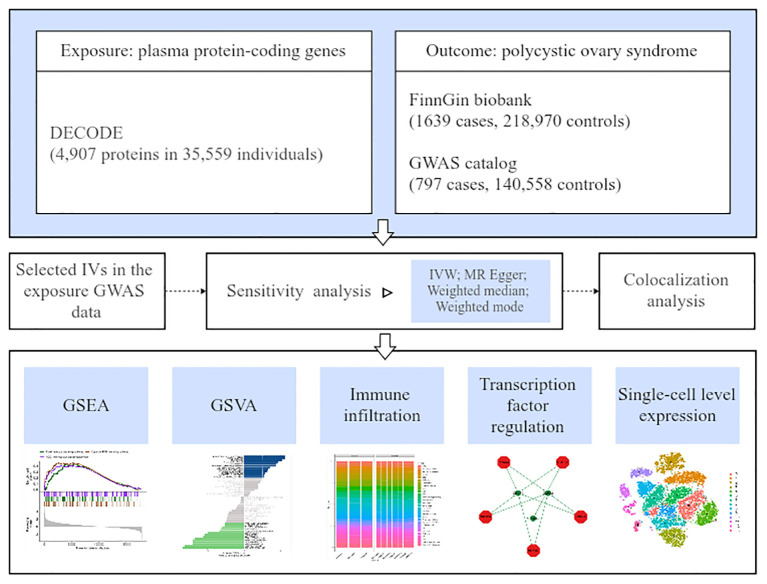
The study design schematic diagram.

**Figure 1** depicts the overall study design.

### Proteome‐wide MR analysis revealing 12 circulating protein-coding genes for PCOS

**Figure 2:**
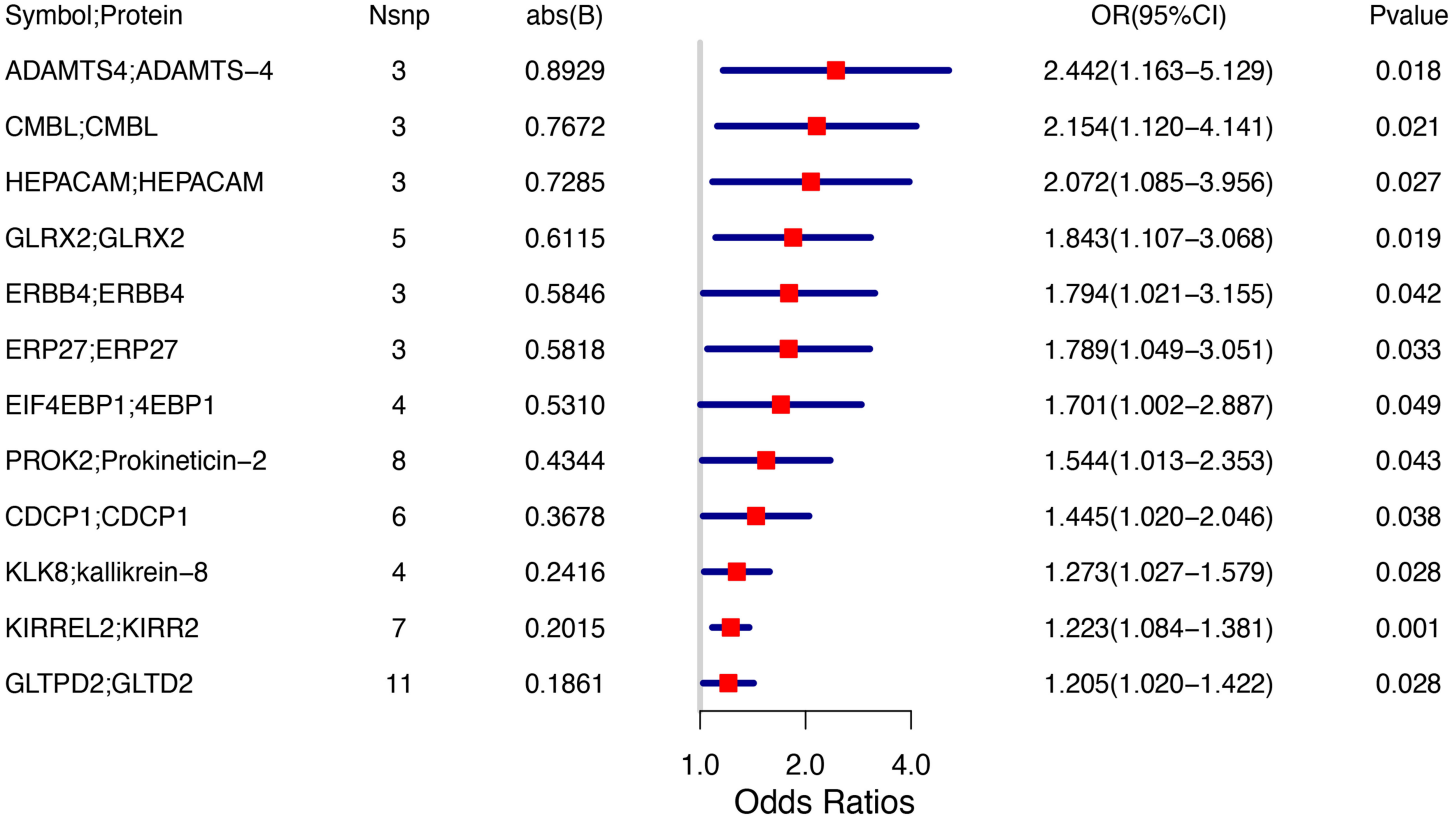
Forest plot indicating Mendelian randomization estimation of the connection between plasma protein-coding genes and PCOS risk.

**Figure 3:**
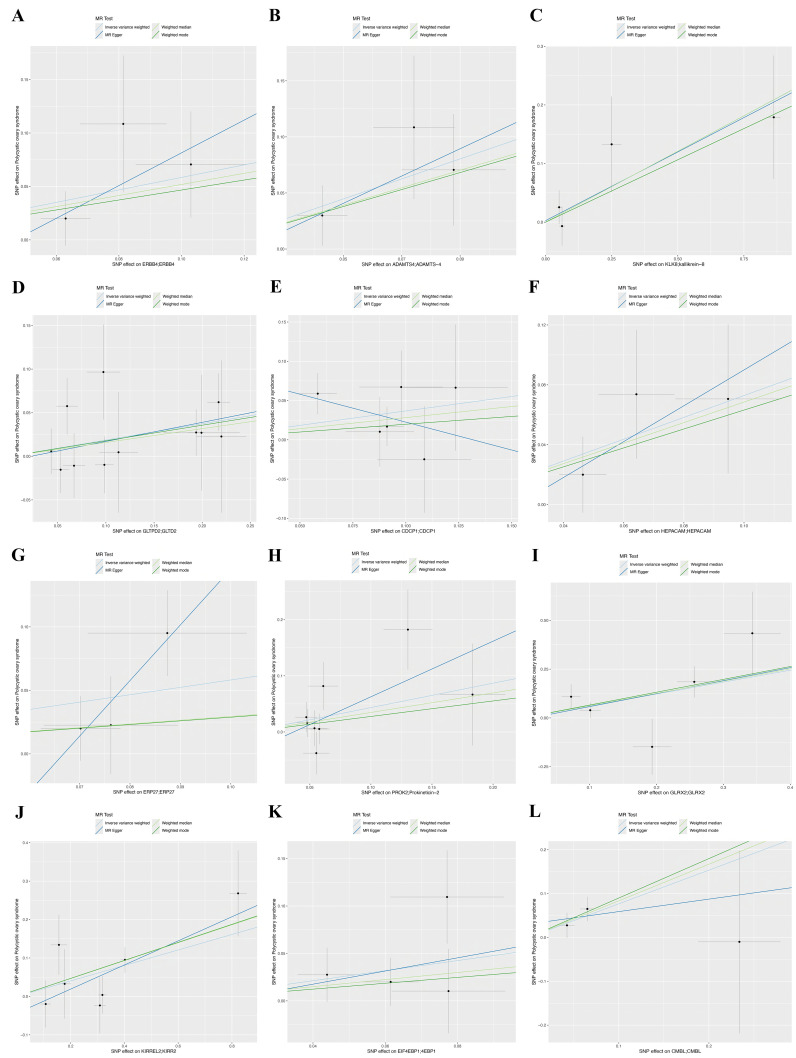
Scatter plots of Mendelian randomization analysis for twelve genes, with distinct colors indicating distinct statistical methods, with the lines’ slopes indicating each method’s causal effect. **(A)** *ERBB4*, **(B)** *ADAMTS4*, **(C)** *KLK8*, **(D)** *GLTPD2*, **(E)** *CDCP1*, **(F)** *HEPACM*, **(G)** *ERP27*, **(H)** *PROK2*, **(I)** *GLRX2*, **(J)** *KIRREL2*, **(K)** *EIF4EBP1*, and **(L)** *CMBL*.

TSMR analysis revealed 243 protein-coding genes causally associated with PCOS. Among the identified proteins, 200 were related to a heightened PCOS risk, whereas the remaining 43 were negatively associated with PCOS risk (**Supplementary Figure 1**). The Q value showed a non-significant *P*-value in the heterogeneity test (*P*>0.05), indicating no heterogeneity in these 243 pairs of causal relationships. In addition, a subsequent leave-one-out analysis showcased that removing a single SNP did not impact causal estimates. To ensure that discrimination was not restricted to one dataset, we used variants from the GWAS Catalog database as another validation dataset. Following causal analysis of these proteins and their association with positive pQTL outcomes, 12 genes were identified as the most significant. These genes include: A disintegrin and metalloproteinase with thrombospondin motifs 4 (*ASAMTS4*), carboxymethylenebutenolidase homolog (*CMBL*), hepatic and glial cell adhesion molecule (*HEPACAM*), glutaredoxin 2 (*GLRX2*), Erb-B2 receptor tyrosine kinase 4 (*ERBB4*), endoplasmic reticulum protein 27 (*ERP27*), eukaryotic translation initiation factor 4E binding protein 1 (*EIF4EBP1*), prokineticin 2 (*PROK2*), CUB domain-containing protein 1 (*CDCP1*), kallikrein-related peptidase 8 (*KLK8*), kirre like nephrin family adhesion molecule 2 (*KIRREL2*), glycolipid transfer protein domain containing 2 (*GLTPD2*), are all related to a heightened PCOS risk (**Figure 2A**). A calculation of OR and 95% confidence interval (95% CI) was carried out. Specifically, *ASAMTS4* (OR=2.442; 95% CI 1.163–5.129; *P*=0.018), *CMBL* (OR=2.154; 95% CI 1.120–4.141; *P*=0.021), *HEPACAM* (OR=2.072 ; 95% CI 1.085–3.956; *P*=0.027), *GLRX2* (OR=1.843; 95% CI 1.107–3.068; *P*=0.019), *ERBB4* (OR=1.794; 95% CI 1.021–3.155; *P*=0.042 ), *ERP27* (OR=1.789 ; 95% CI 1.049–3.051; *P*=0.033), *EIF4EBP1* (OR=1.701 ; 95% CI 1.002–2.887; *P*=0.049), *PROK2* (OR=1.544; 95% CI 1.013–2.353; *P*=0.043 ), *CDCP1*(OR=1.445 ; 95% CI 1.020–2.046; *P*=0.038 ), *KLK8* (OR=1.273; 95% CI 1.027–1.579; *P*=0.028), *KIRREL2* (OR=1.223 ; 95% CI 1.084–1.381; *P*=0.001), and *GLTPD2* (OR=1.205 ; 95% CI 1.020–1.422; *P*=0.028) (**Figure 2**, **Figures 3A–L**). Causal associations between the 12 gene pairs and the pQTL outcomes were rigorously assessed using MR analysis. Additionally, the Q-values derived from the heterogeneity tests exceeded the threshold of 0.05 for all 12 gene pairs, thereby indicating a lack of heterogeneity within these causal relationships (**Table 2**).

**Table 2:** Heterogeneity results from the Cochran’s Q test.

Exposure	Outcome	Method	Q	Q_df	Q_pval
ADAMTS4; ADAMTS-4	PCOS	MR Egger	0.4697	1	0.4931
		IVW	0.5552	2	0.7576
CDCP1; CDCP1	PCOS	MR Egger	2.3202	4	0.6771
		IVW	4.1395	5	0.5295
CMBL; CMBL	PCOS	MR Egger	0.8707	1	0.3508
		IVW	1.1502	2	0.5627
EIF4EBP1; 4EBP1	PCOS	MR Egger	2.6767	2	0.2623
		IVW	2.6933	3	0.4414
ERBB4; ERBB4	PCOS	MR Egger	0.8850	1	0.3468
		IVW	1.3885	2	0.4994
ERP27; ERP27	PCOS	MR Egger	0.2939	1	0.5877
		IVW	2.7060	2	0.2585
GLRX2; GLRX2	PCOS	MR Egger	6.0105	3	0.1111
		IVW	6.0298	4	0.1969
GLTPD2; GLTD2	PCOS	MR Egger	6.7742	9	0.6606
		IVW	6.8321	10	0.7412
HEPACAM; HEPACAM	PCOS	MR Egger	0.5014	1	0.4789
		IVW	0.6868	2	0.7094
KIRREL2; KIRR2	PCOS	MR Egger	5.4461	5	0.3639
		IVW	6.1284	6	0.4090
KLK8; Kallikrein-8	PCOS	MR Egger	1.4912	2	0.4744
		IVW	1.5024	3	0.6817
PROK2; Prokineticin-2	PCOS	MR Egger	7.1368	6	0.3084
		IVW	8.2992	7	0.3070

IVs, instrumental variables; IVW, inverse-variance-weighted; PCOS, polycystic ovary syndrome.

**Figure 4:**
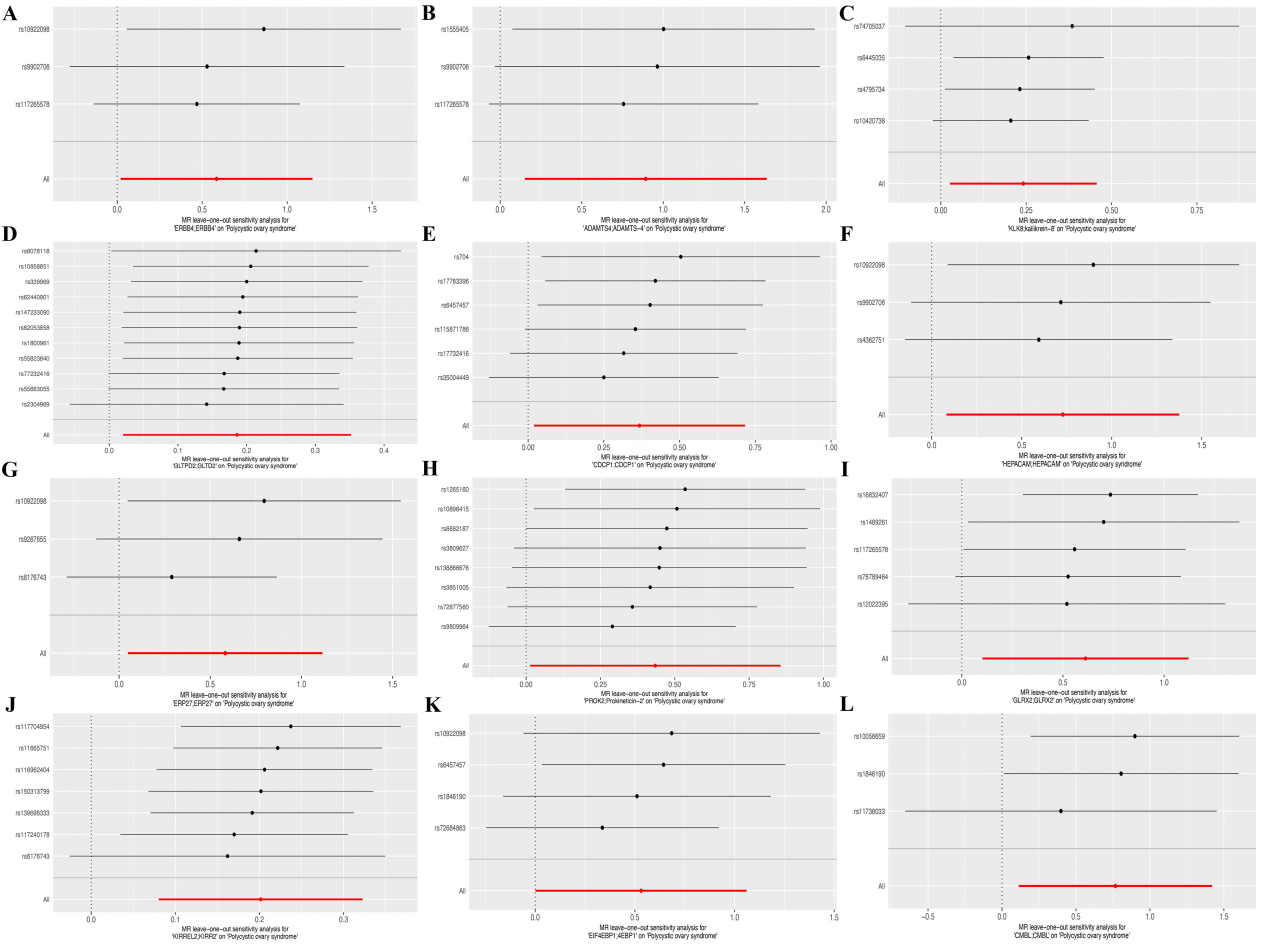
Forest plots of leave-out test for SNPs corresponding to the twelve identified genes. **(A)** *ERBB4*, **(B)** *ADAMTS4*, **(C)** *KLK8*, **(D)** *GLTPD2*, **(E)** *CDCP1*, **(F)** *HEPACM*, **(G)** *ERP27*, **(H)** *PROK2*, **(I)** *GLRX2*, **(J)** *KIRREL2*, **(K)** *EIF4EBP1*, and **(L)** *CMBL*.

Sensitivity analyses demonstrated minimal fluctuations in the overall error margin once excluding any single SNP, suggesting a high degree of robustness in the selected gene pairs (**Figures 4A–L**).

### Co-localization analyses supported the causality of three protein-coding genes with PCOS

**Figure 5:**
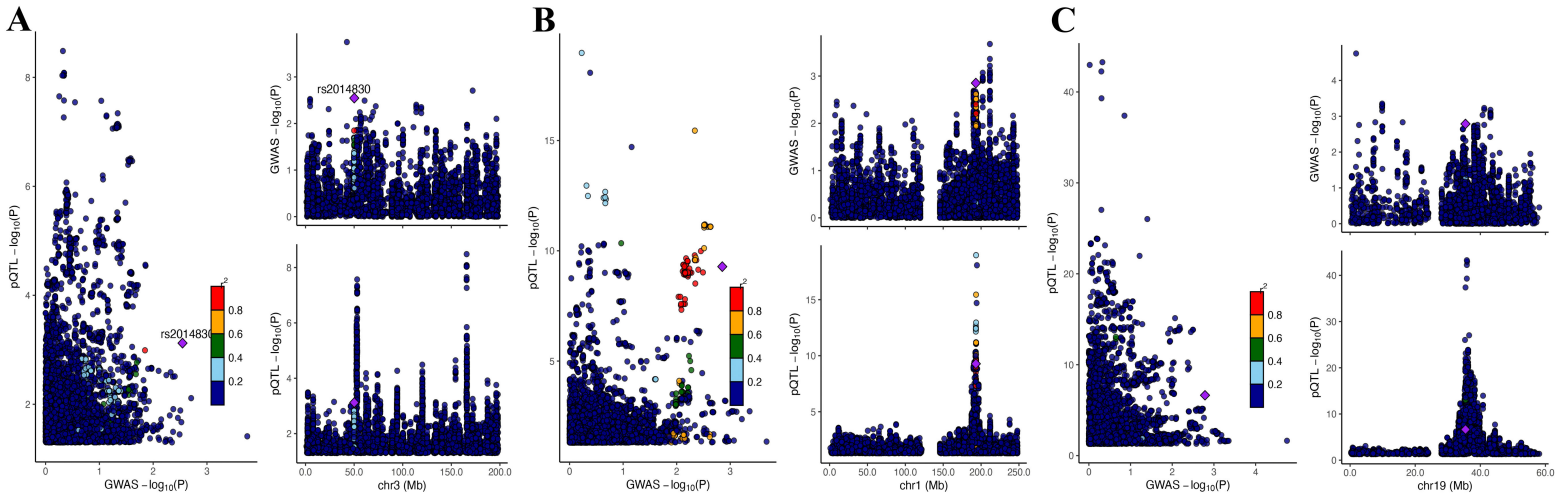
Co-localization analysis. **(A)** *CDCP1*, **(B)** *GLRX2*, and **(C)** *KIRREL2*.

To identify the genes implicated in PCOS, we conducted a co-localization analysis at the pQTL-GWAS interface for the 12 selected genes. Notably, the co-localization of PP.H4 with *CDCP1*, *GLRX2*, and *KIRREL2* exceeded the threshold of 0.65, indicating their significance as target genes for subsequent research (**Figures 5A–C**).

### GSEA and GSVA of signaling pathways for the three genes

**Figure 6:**
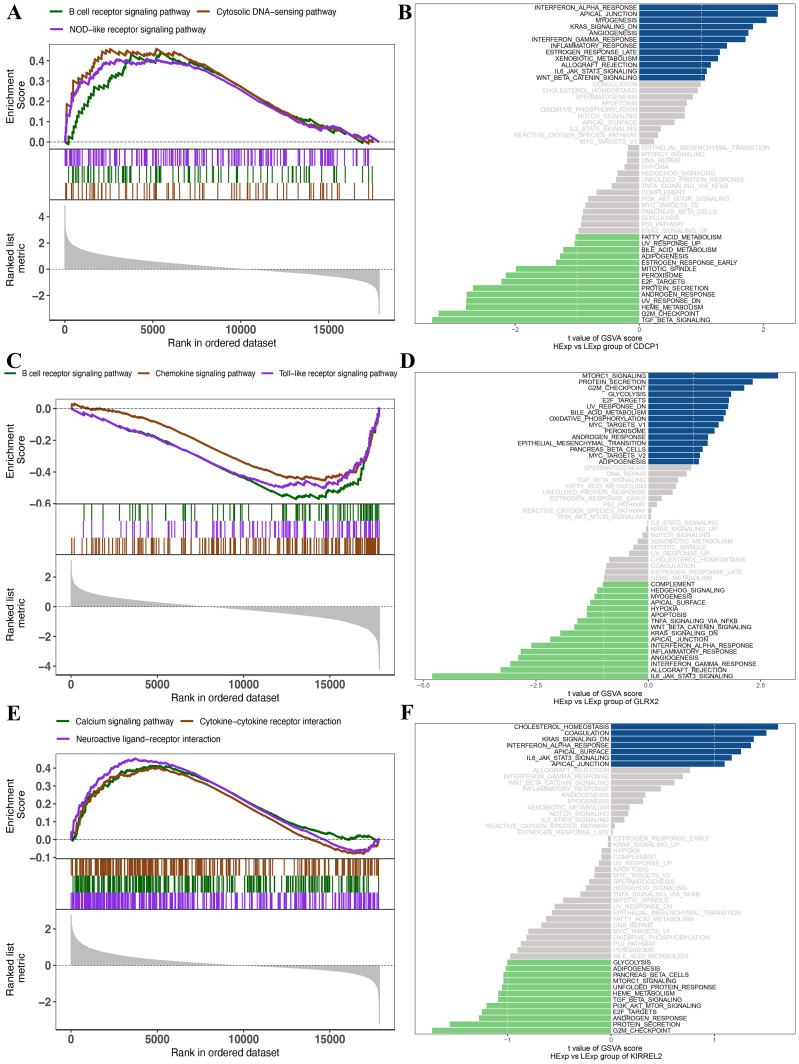
**(A)** GSEA results of *CDCP1*. **(B)** GSVA results of *CDCP1*. **(C)** GSEA results of *GLRX2*. **(D)** GSVA results of *GLRX2*. **(E)** GSEA results of *KIRREL2*. **(F)** GSVA results of *KIRREL2*.

Subsequently, an examination was conducted on the precise pathways related to these three key genes to understand the probable molecular processes through which these genes affect PCOS progression. GSEA findings manifested that *CDCP1* was enriched in various pathways, such as the B-cell receptor, cytosolic DNA-sensing, and NOD-like receptor (**Figure 6A**). *GLRX2* was enriched in pathways including B-cell receptor, chemokine, and Toll-like receptor (**Figure 6B**). KIRREL2 was enriched in calcium signaling, cytokine-cytokine receptor interactions, and neuroactive ligand-receptor interaction pathways (**Figure 6C**). The GSVA results elucidated that these three gene expressions were associated with several signaling pathways. *CDCP1* overexpression enriched ESTROGEN_RESPONSE_LATE and IL6_JAK_STAT3_SIGNALING, while low expression was associated with ESTROGEN_RESPONSE_EARLY and FATTY_ACID_METABOLISM (**Figure 6D**). High expression of *GLRX2* enriched MTORC1_SIGNALING and ANDROGEN_RESPONSE, while low expression was associated with IL6_JAK_STAT3_SIGNALING and INFLAMMATORY_RESPONSE (**Figure 6E**). High expression of *KIRREL2* exhibited main enrichment in CHOLESTEROL_HOMEOSTASIS and IL6_JAK_STAT3_SIGNALING, whereas low expression *KIRREL2* was related to ANDROGEN_RESPONSE and PI3K_AKT_MTOR_SIGNALING (**Figure 6F**).

### Key genes are closely linked to ICI

**Figure 7:**
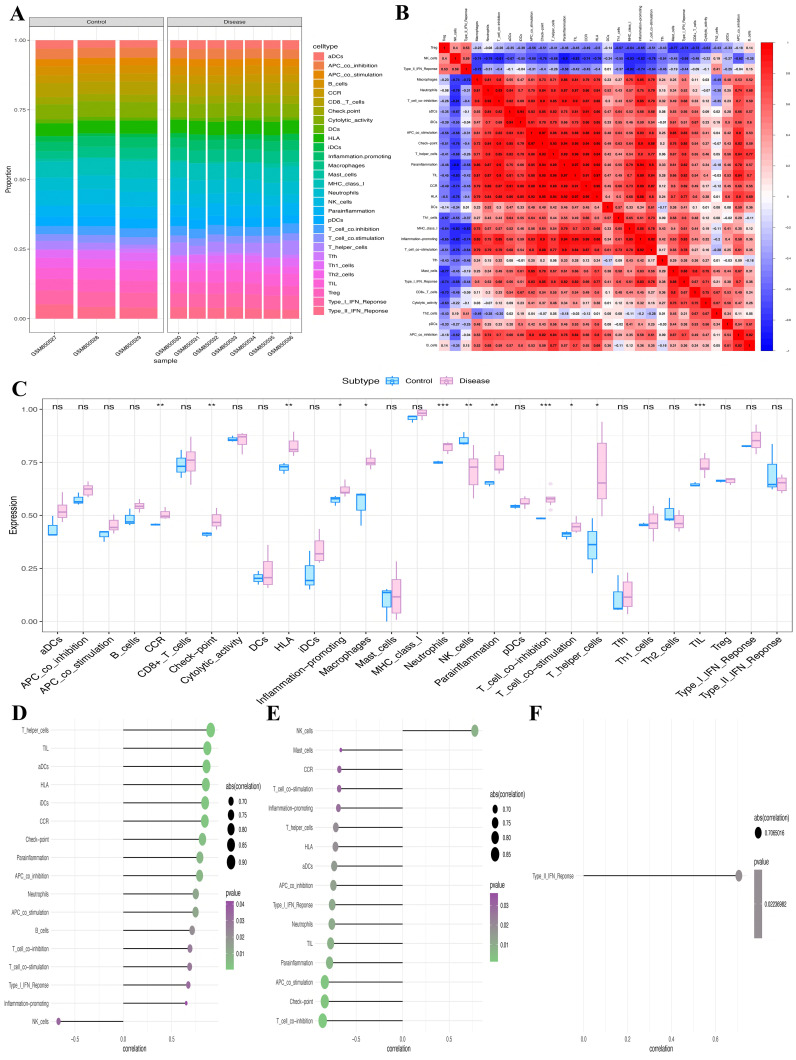
Assessment of immune infiltration. **(A)** Relative infiltrating proportion of 29 immune cell subtypes. **(B)** Correlation relationship between 29 immune cell subtypes. **(C)** Differences in immune cell infiltration between PCOS patients and controls. **(D-F)** The relationship between key genes **(D)** *CDCP1*, **(E)** *GLRX2*, and **(F)** *KIRREL2* and immune cell subtypes (*P<0.05, **P<0.01, ***P<0.001). ns, non-significant.

We conducted an extensive analysis to investigate the molecular processes via which key genes affect PCOS progression. Specifically, we examined the correlation between these key genes and ICI in PCOS. Each individual’s relative immune cell proportion and the correlations among 29 different immune cell subtypes were determined (**Figures 7A, B**), revealing significantly higher numbers of neutrophils, T cell co-inhibition, and macrophages (*P*<0.001, *P*<0.001, *P*<0.05, respectively) than their corresponding levels in the control group (**Figure 7C**). Furthermore, the relation between the key genes and immune cell subtypes was explored. Specifically, *CDCP1* showed significant positive and negative correlations with T_helper_cells and NK_cells, respectively; *GLRX2* demonstrated significant positive and negative correlations with NK_cells and T_cell_co-inhibition, respectively; and *KIRREL2* had a significant positive correlation with Type_II_IFN_ response (**Figures 7D–F**).

### TF regulation

**Figure 8:**
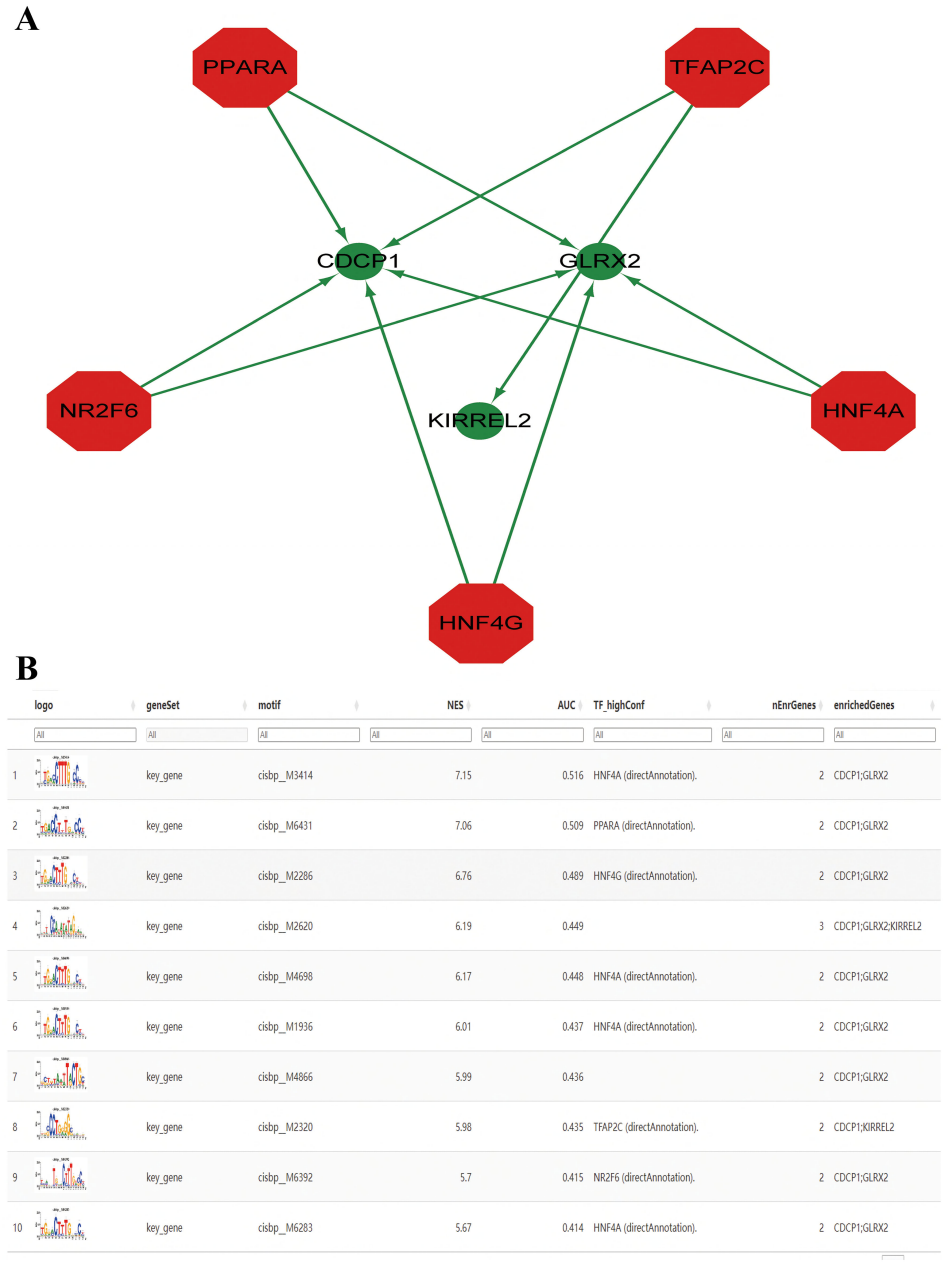
Prediction of transcription factors (TFs). **(A)** The key genes-TF regulatory network. The red node represents TFs, and the green node represents key genes. **(B)** Enrichment analysis of TF binding motifs of the key genes.

Herein, these three key genes were governed by frequent mechanisms involving multiple TFs (**Figure 8A**). Results showcased that the motif having the highest NES (7.15) was cisbp _ M3414. All enriched motifs of the key genes and their corresponding TFs are shown in descending order of their NESs (**Figure 8B**).

### Expression of key genes at the SC level

**Figure 9:**
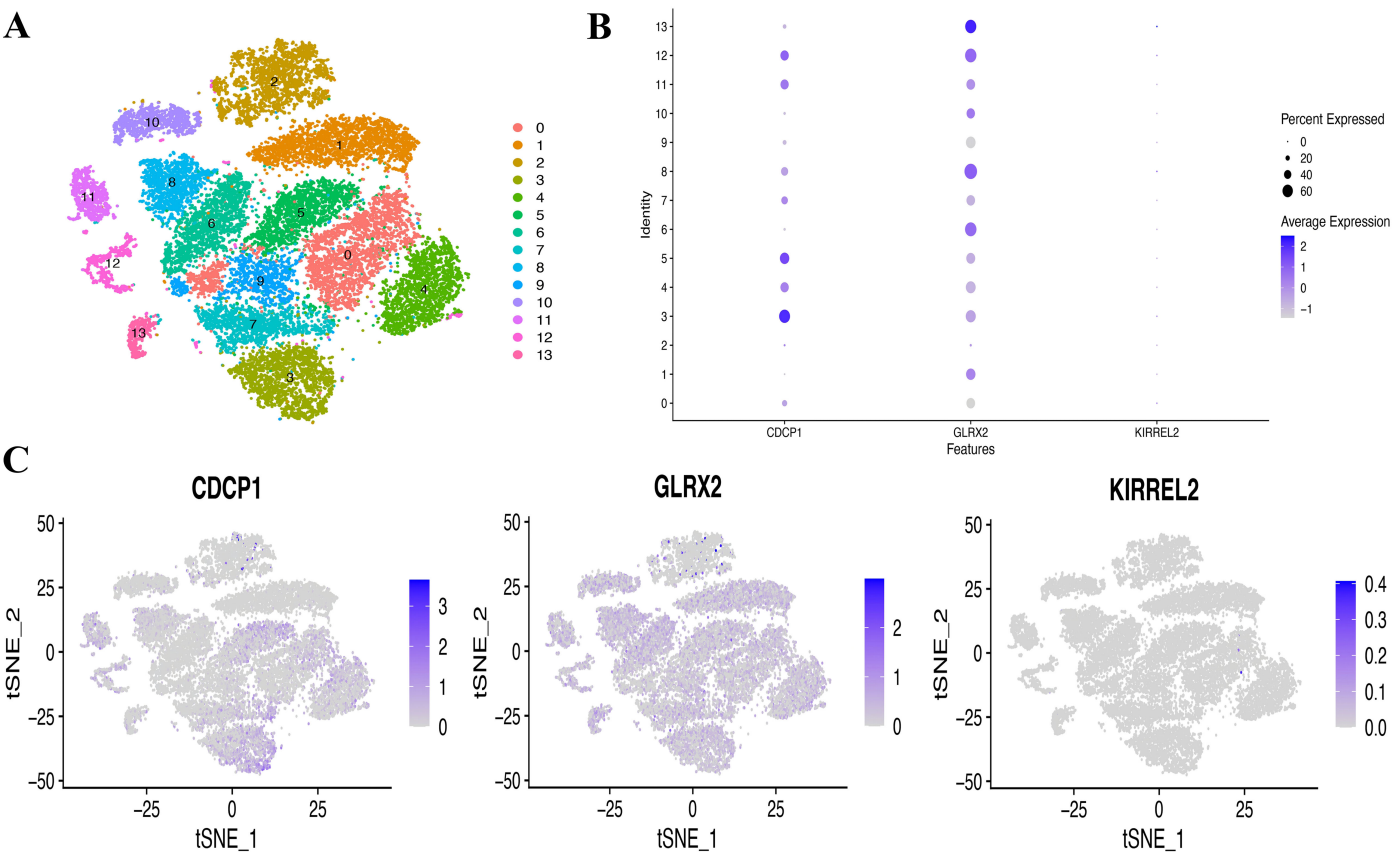
Single-cell (SC) type expression in PCOS for protein-coding genes. **(A)** tSNE cluster analysis diagram. **(B, C)** SC gene expression of three coding genes in every cluster.

**Figure 10:**
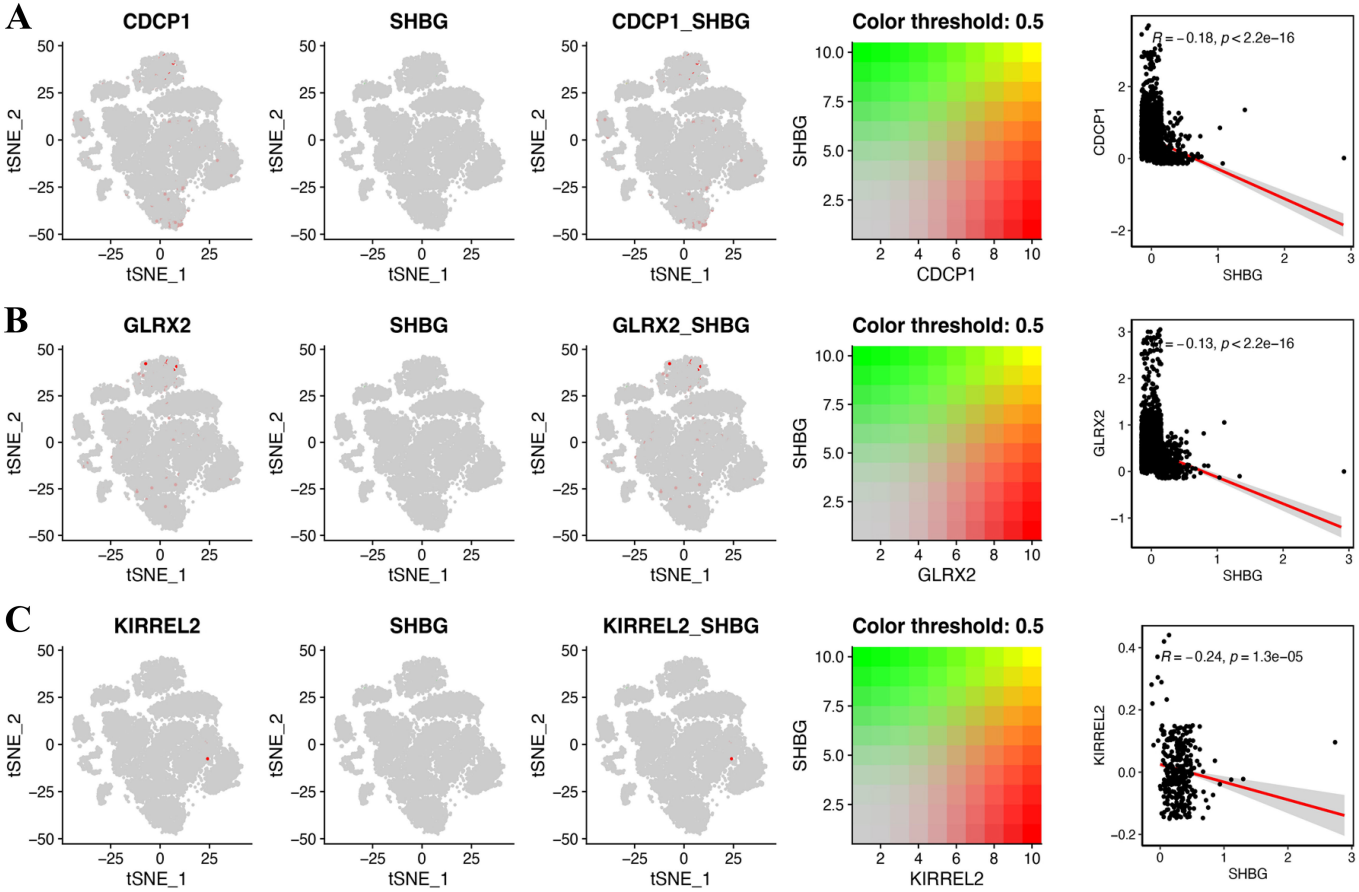
Gene co-expression of SHBG and three key genes at the single cell level. **(A)** *CDCP1*, **(B)** *GLRX2*, and **(C)** *KIRREL2*.

Aiming at exploring whether the coding genes of the three circulating proteins possessed any cell type-specific enrichment in theca cells from the ovaries, we further analyzed scRNA-seq data from the Zenodo database. After clustering using the tSNE algorithm, 14 subtypes of PCOS theca cells were identified (**Figure 9A**). The expression of key genes in these populations was analyzed, revealing that *CDCP1* was overexpressed in subtype 3, and *GLRX2* was highly expressed in subtype 13, whereas the expression of *KIRREL2* was rarely detected (**Figures 9B, C**). In addition, the co-expression of sex hormone-binding globulin (SHBG) and the three key genes in single cells were visualized (**Figures 10A–C**).

## Discussion

According to our best awareness, this study is the first to deploy MR analysis to investigate the causal correlation between plasma protein-coding genes and PCOS. Bioinformatics analyses using SC data were conducted to explore molecular mechanisms further. Employing summary statistics from DECODE genetics, the FinnGen biobank, and the GWAS Catalog database, we found that three protein-coding genes (*CDCP1*, *GLRX2*, and *KIRREL2*) may have causal associations with PCOS risk, which were all associated with increased PCOS susceptibility. Using both RNA-seq (seven cases with PCOS and three controls) and scRNA-seq data from ten individuals, immune infiltration analysis, pathway enrichment analysis, and gene expression levels at the SC level were explored to probe the specific molecular mechanisms behind PCOS.

CDCP1 functions as a transmembrane glycoprotein comprising three extracellular CUB domains (42). Targeting CDCP1 has proved effective in ovarian cancer models (43, 44) and may contribute to cell adhesion and cell-matrix association, which may be related to increased metabolic risks (45). Hatziagelaki et al. enrolled 63 PCOS women in their study, suggesting that CDCP1 expression is negatively correlated with SHBG, which is consistent with our results (46), and the levels of CDCP1 have a relatively strong positive correlation with follicle-stimulating hormone. CDCP1 is one of the most promising targets for therapeutics and diagnostics, reported by many studies of tumors, which were mainly involved in the epithelial-mesenchymal transition process and immune infiltration (42, 47). The available literature demonstrated that CDCP1 might have an immunoregulatory function that is not only relevant to tumor biology, but also potential therapeutic applications in patients with neuroinflammatory conditions and autoimmune endocrine disorders (48, 49). Metabolism of iron and participation in protein glutathionylation/deglutathionylation (50), GLRX2 deficiency has been elucidated to increase oxidative stress cellular sensitivity in metabolic dysfunction-associated fatty liver disease (51). Given the strong association between PCOS and metabolic dysfunction, we speculated that GLRX2 may contribute to PCOS, as oxidative stress is implicated in PCOS pathophysiology. Young et al. showcased that GLRX2 deletion protects mice from diet-triggered weight gain; this phenomenon is associated with heightened mitochondrial respiration and proton leaks (52). Currently, there is no relevant literature on the association between GLRX2 and PCOS; accordingly, further basic and clinical studies should explore this issue. KIRREL2, an immunoglobulin superfamily protein with β-cell-specific expression in the pancreas (53), is encoded by the *KIRREL2* gene in humans. As a regulator of basal insulin secretion in pancreatic cells, KIRREL2 plays an important role (53). In the nonobese diabetic mouse model during the pathogenesis of diabetes, the progressive decline in the expression of KIRREL2 was observed (54). Compared with the other two genes, *KIRREL2* expression levels in the ovarian theca cells of PCOS patients were relatively low in our study. However, no studies have documented the role of KIRREL in PCOS. Therefore, it is advisable to exercise caution while interpreting the results.

The significance of the inflammatory immune mechanism behind PCOS onset and progression has been highlighted, and numerous studies have linked chronic low-grade inflammation to PCOS and have revealed its interaction with it (55, 56). Compared with non-PCOS women, the immune cells such as macrophages, lymphocytes, and natural killer (NK) cells in PCOS patients were increased, and the levels of inflammatory factors such as C-reactive protein, interleukin (IL) 6, IL-18, tumor necrosis factor-*α* and monocyte chemotactic protein-1 in peripheral blood were significantly increased, which makes the ovary and peripheral blood in a state of chronic inflammation (56). Herein, the key gene expression was significantly associated with ICI, including neutrophils, T cell co-inhibition, and macrophages, compared to normal ovulatory women. In this context, the findings from our research align with those outlined by the previously referenced authors. To be specific, we found that CDCP1 expression levels in PCOS had a significant positive correlation with neutrophils, suggesting that the neutrophil count increases accompanied by hyperandrogenism in PCOS, resulting in a chronic low-grade inflammation state (57). However, it was observed that levels of GLRX2 expression in PCOS had a significant negative correlation with neutrophils. Since no previous literature has reported an association between GLRX2 and PCOS and its underlying mechanisms, our negative results should be interpreted with caution. NK cells constitute a significant part of human lymphocyte lineages, making up approximately 10-15% of lymphocytes in circulation (58). It is demonstrated that a substantial increase was observed in NK cells in infertile women with PCOS, indicating that the active state of NK cells may promote ovarian fibrosis and affect follicle survival, resulting in rare ovulation or anovulation, reported by He et al. (59). In our research, NK cells were negative with the expression of CDCP1 but positive with GLRX2 expression. Due to the correlation analysis between the expression level of key genes and the level of immune cells performed using transcriptome data with a relatively small sample size, additional study is warranted to explore the role of these protein-coding genes in NK cell functions. T lymphocytes are crucial in the local PCOS pathological mechanisms, as reported by Wu et al. (28). T cells are categorized into three subsets: helper, cytotoxic, and Treg cells. The Th1/Th2 imbalance is associated with IR, hyperandrogenism, and polycystic ovarian changes in PCOS, as well as the reduction of chances of pregnancy and increased risk of abortion (60). Besides, reduced peripheral blood Treg cell levels contribute to lower anti-inflammatory factor levels in women with PCOS (61). Notably, both CDCP1 and GLRX2 were enriched in the B-cell receptor pathway according to our results. The activation of B cells could promote the development of PCOS-like metabolic phenotype in mice (31). Perhaps further analysis should investigate how the coding genes affect the PCOS phenotype via B cells and B-cell-related pathways. Additionally, identifying and regulating the state of macrophage polarization may provide new diagnostic and therapeutic strategies for PCOS (62). Macrophage polarization and inflammatory cytokines secretion by macrophages can cause systemic chronic low-grade inflammation in PCOS patients (63, 64). In our study, differences in macrophage infiltration were observed between patients with PCOS and controls; however, no correlations between the three genes and macrophages were detected.

Our strength point is that we are the first to report the causal relationship between plasma proteins and their coding genes and PCOS risk and explore the molecular mechanisms behind the MR and bioinformatics analysis integration. Our study has a few limitations that cannot be overlooked. First, four phenotypes (phenotypes A, B, C, and D) of PCOS relying upon the diagnostic criteria were analyzed together in the GWAS; nevertheless, we could not perform MR targeting a certain phenotype, and only an overall analysis of PCOS was allowed. Approximately 60% of the patients included in PCOS GWASs have been projected to exhibit a single phenotype (phenotype A) (65). For further analysis, hormonal levels of selected PCOS patients should meet the criteria that the level of total testosterone is higher than 0.5ng/mL (66). Meanwhile, patients with comorbidities such as prediabetes or 17α-dihydroxy-progesterone > 2ng/mL should be excluded. We expect that in the coming years, a large GWAS will specifically dissect these phenotypes. Second, it should be noted that the participants in the original MRI data of MR are exclusively from Europe, which may limit the generalizability of the results to individuals from other continents or with other ancestries. Third, different tissues may have distinct genetic regulatory mechanisms, and an understanding of PCOS based solely on blood pQTLs may not be comprehensive. In addition, scRNA-seq data were obtained from ovarian theca cells, and due to the heterogeneity of PCOS, analysis of other tissues may facilitate our understanding of the pathogenesis of PCOS as well since some ethical concerns must be considered. Perhaps performing scRNA-seq analysis of different organs of PCOS rodent models is needed in the future. In our analysis, the correlation analysis between the expression level of protein-coding genes and the level of immune infiltration was performed using the transcriptome data rather than the scRNA-seq data. The pivotal of tailor-made therapy for PCOS with high heterogeneity was acknowledged. Therefore, appropriately designed clinical trials (randomized clinical trials) with larger sample sizes containing five groups (healthy controls and phenotypes A, B, C, and D, respectively) are needed before they can be suitable for daily clinical therapeutic practice. Parameters related to glucose and lipid metabolism, the extent of inflammation, hyperandrogenism, menstrual cycle, anthropometrics, etc. need to be recorded. Additionally, adverse events in the process of therapy cannot be ignored (67, 68).

## Conclusion

In summary, through the integration of genetics and the human plasma proteome, we found that the levels of three protein-coding genes (*CDCP1*, *GLRX2*, and *KIRREL2*) may be linked to a higher risk of PCOS, suggesting that they may be an entry point for exploring the pathogenesis and treatment of PCOS, warranting further basic and clinical investigations.

## Data Availability

Publicly available datasets were analyzed in this study. This data can be found here: the single cell data (https://zenodo.org/record/7942968) from the Zenodo database, the transcriptome data (https://www.ncbi.nlm.nih.gov/geo/query/acc.cgi?acc=GSE34526) from the GEO database, the plasma protein (https://www.decode.com/summarydata/) from the DECODE database, outcome data sources (https://r10.finngen.fi/) from the FinnGen consortium R10 release and (https://www.ebi.ac.uk/gwas/studies/GCST90044902) from the GWAS Catalog database. The above data are publicly available, and further inquiries including analysis coding can be directed to the corresponding authors.

## Supplementary material

Supplementary Figure 1 The causality analysis result of the two-sample Mendelian randomization analysis shows 243 gene pairs for PCOS risk relationships.
