# Supplementary material for: From proteome to pathogenesis: investigating polycystic ovary syndrome with Mendelian randomization analysis
Source: Front Endocrinol (Lausanne). 2024 Sep 9;15:1442483. doi: 10.3389/fendo.2024.1442483 (PMC11417002; doi:10.3389/fendo.2024.1442483)

Symbol;Protein	Nsnp	abs(B)	OR(95%CI)	Pvalue
PLS1;PLSI	3	1.6183	5.045(1.656−15.366)	0.004
PLBD1;PLBL1	6	1.3867	4.002(2.009−7.971)	<0.001
APBB2;APBB2	4	1.3718	3.942(1.629−9.541)	0.002
MAGEA4;MAGE−4	3	1.2974	3.660(1.016−13.184)	0.047
TRH;TRH	3	1.2816	3.602(1.483−8.753)	0.005
APPL1;DP13A	6	1.2811	3.601(1.621−7.996)	0.002
HSPH1;HS105	3	1.2142	3.368(1.045−10.854)	0.042
HNF1A;HNF1A	4	1.1737	3.234(1.058−9.885)	0.040
CMBL;CMBL	3	1.1393	3.124(1.262−7.736)	0.014
HEPACAM;HEPACAM	4	1.0800	2.945(1.114−7.782)	0.029
IGFBP4;IGFBP−4	4	1.0783	2.940(1.468−5.885)	0.002
ELL2;ELL2	5	1.0691	2.913(1.321−6.422)	0.008
IL10;IL−10	6	1.0547	2.871(1.304−6.321)	0.009
ASF1A;ASF1A	5	1.0459	2.846(1.150−7.044)	0.024
SETD2;SETD2	5	0.9975	2.711(1.168−6.297)	0.020
TOPBP1;TOPB1	4	0.9811	2.667(1.012−7.030)	0.047
TIMM21;TIM21	3	0.9748	2.651(1.027−6.839)	0.044
CARD17;CAR17	3	0.9570	2.604(1.175−5.769)	0.018
SDHAF2;SDHF2	3	0.9476	2.579(1.006−6.614)	0.049
SENP7;SENP7	4	0.9466	2.577(1.006−6.604)	0.049
SLC30A5;ZNT5	4	0.9286	2.531(1.211−5.291)	0.014
ZNF329;ZN329	5	0.9266	2.526(1.249−5.108)	0.010
PTPN13;PTN13	5	0.9112	2.487(1.293−4.786)	0.006
FEV;FEV	5	0.8879	2.430(1.065−5.544)	0.035
DYNLL1;DLC8	6	0.8786	2.407(1.006−5.760)	0.048
FAM19A2;F19A2	4	0.8780	2.406(1.214−4.769)	0.012
DPYSL3;DPYL3	4	0.8770	2.404(1.102−5.242)	0.028
POLI;POLI	7	0.8751	2.399(1.304−4.415)	0.005
GDNF;GDNF	4	0.8699	2.387(1.237−4.606)	0.010
HNRNPR;HNRPR	5	0.8671	2.380(1.177−4.814)	0.016
DPYSL5;DPYL5	7	0.8670	2.380(1.243−4.557)	0.009
ERP27;ERP27	3	0.8596	2.362(1.011−5.521)	0.047
ACAP2;CENB2	5	0.8567	2.355(1.155−4.802)	0.018
ADAMTS4;ADAMTS−4	5	0.8462	2.331(1.075−5.052)	0.032
PTPN1;PTP−1B	5	0.8396	2.315(1.097−4.888)	0.028
ADAM32;ADA32	3	0.8347	2.304(1.041−5.101)	0.040
PRPF6;PRP6	5	0.8312	2.296(1.311−4.020)	0.004
ERBB4;ERBB4	4	0.8280	2.289(1.112−4.710)	0.025
ANPEP;Aminopeptidase−N	6	0.8252	2.282(1.225−4.252)	0.009
SNRPD3;SMD3	4	0.8214	2.274(1.275−4.055)	0.005
EIF4EBP1;4EBP1	5	0.8182	2.267(1.172−4.382)	0.015
PKD2;PKD2	7	0.8180	2.266(1.369−3.750)	0.001
COL6A5;CO6A5	6	0.8163	2.262(1.061−4.821)	0.034
LAMA4;LAMA4	6	0.8092	2.246(1.062−4.751)	0.034
WBP2;WBP2	6	0.7933	2.211(1.040−4.698)	0.039
PRR27;CD040	6	0.7921	2.208(1.370−3.559)	0.001
S100A5;S100A5	3	0.7839	2.190(1.106−4.338)	0.025
HCAR2;HCAR2	6	0.7800	2.181(1.230−3.867)	0.008
CTCF;CTCF	5	0.7775	2.176(1.026−4.613)	0.043
TLR1;TLR1	7	0.7770	2.175(1.117−4.237)	0.022
SERPINB4;SCCA2	6	0.7652	2.149(1.241−3.724)	0.006
KIAA1467;K1467	4	0.7639	2.147(1.073−4.295)	0.031
G6PD;G6PD	5	0.7616	2.142(1.020−4.498)	0.044
BAI3;BAI3	6	0.7608	2.140(1.104−4.149)	0.024
TACSTD2;GA733−1−protein	4	0.7608	2.140(1.122−4.080)	0.021
CDHR5;MUCDL	7	0.7594	2.137(1.259−3.628)	0.005
HDGFL1;HDGL1	4	0.7504	2.118(1.149−3.905)	0.016
MRRF;RRFM	5	0.7474	2.112(1.044−4.271)	0.038
DTYMK;KTHY	7	0.7472	2.111(1.155−3.859)	0.015
DEFB106A;D106A	8	0.7468	2.110(1.242−3.585)	0.006
ENOX2;ENOX2	6	0.7457	2.108(1.071−4.147)	0.031
PANK3;PANK3	4	0.7457	2.108(1.004−4.426)	0.049
CDCP1;CDCP1	8	0.7428	2.102(1.351−3.270)	<0.001
RRAS;RRAS	6	0.7414	2.099(1.384−3.183)	<0.001
TNRC6B;TNR6B	6	0.7409	2.098(1.005−4.378)	0.048
PFDN1;PFD1	4	0.7359	2.087(1.120−3.892)	0.021
ABLIM3;ABLM3	3	0.7335	2.082(1.079−4.018)	0.029
MMP16;MMP−16	6	0.7282	2.071(1.081−3.967)	0.028
PAK4;PAK4	5	0.7268	2.068(1.042−4.106)	0.038
KMT2C;KMT2C	4	0.7265	2.068(1.025−4.172)	0.042
TXNDC12;TXD12	5	0.7146	2.043(1.060−3.938)	0.033
ST6GALNAC6;SIA7F	10	0.7099	2.034(1.136−3.641)	0.017
OIT3;OIT3	6	0.6945	2.003(1.176−3.409)	0.011
GLRX3;GLRX3	3	0.6892	1.992(1.015−3.912)	0.045
CD79B;CD79B	5	0.6870	1.988(1.164−3.394)	0.012
CD44;CD44	5	0.6861	1.986(1.088−3.623)	0.025
CGA;Glycoprotein−hormones−a−chain	3	0.6817	1.977(1.013−3.859)	0.046
EWSR1;EWS	7	0.6806	1.975(1.135−3.436)	0.016
SCAMP5;SCAM5	5	0.6770	1.968(1.029−3.765)	0.041
ASAH1;Acid−ceramidase	6	0.6694	1.953(1.142−3.340)	0.015
DOCK9;DOCK9	4	0.6688	1.952(1.021−3.733)	0.043
SSRP1;SSRP1	7	0.6472	1.910(1.026−3.557)	0.041
ARHGAP36;RHG36	6	0.6345	1.886(1.283−2.773)	0.001
AKR1C1;Aldo−keto−reductase−1C1	6	0.6246	1.867(1.055−3.306)	0.032
IL2;IL−2	5	0.6210	1.861(1.056−3.279)	0.032
F9;Coagulation−Factor−IXab	6	0.6198	1.859(1.082−3.193)	0.025
IL17RB;IL−17B−R	6	0.6185	1.856(1.077−3.199)	0.026
SORBS3;Vinexin−b	7	0.6183	1.856(1.025−3.359)	0.041
LYSMD3;LYSM3	5	0.6136	1.847(1.001−3.408)	0.050
TBC1D5;TBCD5	8	0.6003	1.823(1.029−3.230)	0.040
CETN2;CETN2	8	0.5957	1.814(1.077−3.056)	0.025
MAX;MAX	5	0.5907	1.805(1.065−3.061)	0.028
GNAI3;GNAI3	5	0.5902	1.804(1.076−3.027)	0.025
F9;Coagulation−Factor−IX	7	0.5831	1.792(1.039−3.089)	0.036
WFDC3;WFDC3	7	0.5791	1.785(1.066−2.988)	0.028
C5;C5	10	0.5770	1.781(1.217−2.606)	0.003
LCAT;LCAT	7	0.5743	1.776(1.120−2.817)	0.015
FIBP;FIBP	6	0.5721	1.772(1.024−3.067)	0.041
MAP2K6;MP2K6	9	0.5691	1.767(1.100−2.836)	0.018
RHOB;RHOB	8	0.5645	1.759(1.094−2.828)	0.020
HERC4;HERC4	10	0.5635	1.757(1.220−2.529)	0.002
ENTPD6;ENTP6	6	0.5593	1.750(1.119−2.736)	0.014
ULK3;ULK3	6	0.5446	1.724(1.066−2.787)	0.026
AIMP1;EMAP−2	8	0.5444	1.724(1.114−2.666)	0.014
IFNA16;IFN16	9	0.5324	1.703(1.053−2.755)	0.030
SPHK2;SPHK2	8	0.5268	1.693(1.056−2.716)	0.029
PQBP1;PQBP1	6	0.5134	1.671(1.026−2.723)	0.039
USH1C;USH1C	10	0.5113	1.668(1.038−2.679)	0.035
TFRC;TR	14	0.5108	1.667(1.175−2.363)	0.004
SDC3;SDC3	6	0.5103	1.666(1.100−2.523)	0.016
CNTF;CNTF	8	0.5074	1.661(1.079−2.556)	0.021
SUOX;SUOX	7	0.5066	1.660(1.001−2.752)	0.050
EFS;EFS	12	0.4992	1.647(1.204−2.254)	0.002
GIMAP6;GIMA6	7	0.4967	1.643(1.038−2.601)	0.034
UBE2D3;UB2D3	7	0.4951	1.641(1.085−2.480)	0.019
FAM213A;CJ058	10	0.4905	1.633(1.124−2.373)	0.010
MYZAP;MYZAP	7	0.4897	1.632(1.037−2.568)	0.034
HSD17B8;DHB8	5	0.4893	1.631(1.016−2.619)	0.043
NDRG4;NDRG4	6	0.4857	1.625(1.052−2.511)	0.029
MSH2;MSH2	10	0.4857	1.625(1.073−2.462)	0.022
ZP4;ZP4	10	0.4849	1.624(1.011−2.608)	0.045
CASS4;CT032	8	0.4803	1.617(1.027−2.546)	0.038
SH2D1B;SH21B	7	0.4800	1.616(1.125−2.320)	0.009
TGM4;TGM4	6	0.4735	1.606(1.165−2.213)	0.004
ARHGEF2;ARHG2	8	0.4694	1.599(1.015−2.520)	0.043
MAGEA8;MAGA8	6	0.4675	1.596(1.008−2.527)	0.046
THPO;Tpo	6	0.4656	1.593(1.007−2.520)	0.047
LMNB1;Lamin−B1	6	0.4650	1.592(1.043−2.430)	0.031
NEO1;NEO1	12	0.4649	1.592(1.174−2.159)	0.003
TF;Transferrin	3	0.4588	1.582(1.006−2.488)	0.047
APOC1;Apo−C−I	8	0.4566	1.579(1.040−2.396)	0.032
INHBA;Inhibin−bA−chain	10	0.4550	1.576(1.073−2.315)	0.020
HEPHL1;HPHL1	10	0.4541	1.575(1.111−2.232)	0.011
OLFM3;NOE3	8	0.4516	1.571(1.010−2.442)	0.045
KIR2DS4;KI2S4	10	0.4512	1.570(1.066−2.314)	0.023
ENPP6;ENPP6	7	0.4507	1.569(1.027−2.397)	0.037
SKIL;SKIL	9	0.4502	1.569(1.016−2.421)	0.042
CD37;CD37	7	0.4332	1.542(1.025−2.320)	0.038
TNFAIP8;TFIP8	8	0.4291	1.536(1.035−2.279)	0.033
PFDN5;PFD5	5	0.4266	1.532(1.015−2.311)	0.042
NRBP1;NRBP	9	0.4264	1.532(1.069−2.196)	0.020
NXPH3;NXPH3	12	0.4256	1.531(1.122−2.088)	0.007
EGFL6;EGFL6	12	0.4164	1.517(1.038−2.215)	0.031
FSTL4;FSTL4	8	0.4108	1.508(1.009−2.254)	0.045
ATP5J2;ATPK	6	0.4052	1.500(1.035−2.174)	0.032
CHIA;CHIA	5	0.4026	1.496(1.031−2.170)	0.034
CCL11;Eotaxin	16	0.3965	1.487(1.175−1.880)	<0.001
MFGE8;MFGM	20	0.3904	1.478(1.183−1.846)	<0.001
STAT3;STAT3	7	0.3900	1.477(1.015−2.150)	0.042
EFHD1;EFHD1	9	0.3898	1.477(1.000−2.180)	0.050
ZNF41;ZNF41	4	0.3812	1.464(1.084−1.977)	0.013
FAM20A;FA20A	11	0.3812	1.464(1.073−1.998)	0.016
GLRX2;GLRX2	10	0.3734	1.453(1.045−2.019)	0.026
NAE1;ULA1	9	0.3522	1.422(1.021−1.981)	0.037
CPE;CBPE	12	0.3417	1.407(1.002−1.976)	0.049
RSPO3;RSPO3	11	0.3396	1.404(1.097−1.797)	0.007
ERCC1;ERCC1	6	0.3270	1.387(1.034−1.860)	0.029
PI3;Elafin	12	0.3270	1.387(1.087−1.769)	0.009
ITPRIPL1;IPIL1	10	0.3186	1.375(1.051−1.799)	0.020
FBXL4;FBXL4	9	0.3160	1.372(1.004−1.874)	0.047
HS6ST1;H6ST1	15	0.3145	1.370(1.027−1.827)	0.032
KLK8;kallikrein−8	9	0.3144	1.369(1.016−1.845)	0.039
MASP1;MASP3	11	0.3101	1.364(1.072−1.734)	0.011
RAB3A;Rab3A	10	0.3091	1.362(1.028−1.806)	0.032
PAXIP1;PAXI1	8	0.3077	1.360(1.001−1.848)	0.049
IL1RN;IL−1Ra	11	0.3041	1.355(1.000−1.837)	0.050
TPD52L1;TPD53	10	0.2951	1.343(1.061−1.701)	0.014
APOD;Apo−D	10	0.2949	1.343(1.095−1.648)	0.005
SIGLEC12;SIG12	14	0.2924	1.340(1.060−1.693)	0.014
PDGFC;PDGF−CC	7	0.2919	1.339(1.010−1.774)	0.042
C10orf54;GI24	7	0.2908	1.338(1.007−1.777)	0.045
KIF3A;KIF3A	16	0.2896	1.336(1.003−1.779)	0.048
VSIG2;VSIG2	4	0.2792	1.322(1.065−1.641)	0.011
NPL;NPL	12	0.2713	1.312(1.031−1.669)	0.027
CFB;Factor−B	12	0.2676	1.307(1.105−1.545)	0.002
DHFR;DYR	11	0.2674	1.307(1.028−1.661)	0.029
VWC2;VWC2	17	0.2656	1.304(1.069−1.592)	0.009
GOSR2;GOSR2	11	0.2549	1.290(1.035−1.609)	0.024
DCUN1D5;DCNL5	17	0.2521	1.287(1.054−1.571)	0.013
POMC;Corticotropin−lipotropin	21	0.2509	1.285(1.025−1.612)	0.030
CDSN;CDSN	15	0.2364	1.267(1.024−1.567)	0.029
CD300C;CLM6	23	0.2347	1.265(1.069−1.496)	0.006
KNG1;Kininogen−−HMW−−Two−Chain	12	0.2320	1.261(1.061−1.499)	0.009
GLTPD2;GLTD2	19	0.2291	1.258(1.018−1.553)	0.033
CTRB1;Chymotrypsin	23	0.2222	1.249(1.022−1.525)	0.029
LRTM2;LRTM2	10	0.2147	1.240(1.007−1.525)	0.043
IDUA;IDUA	22	0.2118	1.236(1.068−1.430)	0.005
APOA5;Apo−A−V	23	0.2086	1.232(1.002−1.515)	0.048
FCN3;Ficolin−3	21	0.1984	1.219(1.014−1.467)	0.035
PAF;PAF	20	0.1979	1.219(1.051−1.413)	0.009
BIRC2;cIAP−1	8	0.1823	1.200(1.031−1.397)	0.019
CTSL;Cathepsin−L	8	0.1765	1.193(1.008−1.412)	0.040
APOC3;Apo−C−III	37	0.1741	1.190(1.007−1.407)	0.042
PLA2R1;PLA2R	33	0.1536	1.166(1.007−1.350)	0.040
HTATIP2;HTAI2	14	0.1488	1.160(1.002−1.344)	0.047
GOLM1;GOLM1	19	0.1411	1.151(1.016−1.306)	0.028
HGFAC;HGFA	12	0.1337	1.143(1.007−1.297)	0.038
KIRREL2;KIRR2	20	0.1272	1.136(1.022−1.262)	0.018
CDH5;Cadherin−5	19	0.1129	1.119(1.008−1.244)	0.035
HDGFRP3;HDGR3	12	0.1112	1.118(1.004−1.244)	0.043
ANGPTL3;ANGL3	15	0.1033	1.109(1.024−1.201)	0.011
FOLR3;FOLR3	23	0.0926	0.912(0.843−0.986)	0.021
SOD3;SOD3	19	0.1061	0.899(0.810−0.999)	0.048
TST;THTR	41	0.1540	0.857(0.748−0.983)	0.027
LILRA6;LIRA6	12	0.1551	0.856(0.768−0.955)	0.005
NAAA;ASAHL	40	0.1561	0.856(0.759−0.965)	0.011
LILRA4;LIRA4	22	0.1594	0.853(0.731−0.994)	0.042
TP53I3;QORX	15	0.1650	0.848(0.721−0.997)	0.046
CBLN1;CBLN1	41	0.1667	0.846(0.720−0.995)	0.044
FCGR2A;FCG2A	12	0.1762	0.838(0.744−0.945)	0.004
PMEL;GP100	25	0.1819	0.834(0.698−0.995)	0.044
QPCT;Glutaminyl−cyclase	32	0.1979	0.820(0.681−0.988)	0.037
LARGE;LARGE	26	0.1991	0.819(0.675−0.995)	0.044
TREML2;TRML2	11	0.2044	0.815(0.680−0.978)	0.027
ACP1;PPAC	10	0.2046	0.815(0.705−0.942)	0.006
SIRT2;SIRT2	18	0.2252	0.798(0.673−0.947)	0.010
FAS;Fas−−soluble	10	0.2293	0.795(0.635−0.995)	0.045
PROK2;Prokineticin−2	18	0.2340	0.791(0.628−0.997)	0.047
CD248;CD248	25	0.2424	0.785(0.621−0.992)	0.043
CLSTN2;CSTN2	19	0.2535	0.776(0.610−0.988)	0.039
MYL3;Myosin−light−chain−1	20	0.2979	0.742(0.582−0.947)	0.016
CD55;DAF	19	0.3178	0.728(0.596−0.888)	0.002
CCL5;RANTES	18	0.3223	0.724(0.533−0.986)	0.040
BMP7;BMP−7	14	0.3372	0.714(0.518−0.983)	0.039
GFRA2;GFRa−2	18	0.3481	0.706(0.510−0.978)	0.036
GSN;Gelsolin	8	0.3803	0.684(0.502−0.932)	0.016
BCAN;PGCB	6	0.3847	0.681(0.496−0.935)	0.017
FCGR1A;FCGR1	4	0.3947	0.674(0.456−0.997)	0.048
IGF2;IGF−II	11	0.4000	0.670(0.487−0.923)	0.014
HGF;HGF	9	0.4313	0.650(0.453−0.931)	0.019
BDNF;BDNF	10	0.4793	0.619(0.405−0.946)	0.027
SELM;SELM	14	0.5023	0.605(0.414−0.884)	0.009
PDYN;Dynorphin−A−−1−17	8	0.5272	0.590(0.428−0.814)	0.001
TPPP2;TPPP2	12	0.5432	0.581(0.372−0.906)	0.017
RHOD;RHOD	5	0.5672	0.567(0.363−0.887)	0.013
DEFB4A;HBD−2	11	0.5925	0.553(0.345−0.886)	0.014
TBXAS1;THAS	6	0.6365	0.529(0.329−0.850)	0.009
ROBO2;ROBO2	11	0.6694	0.512(0.350−0.749)	<0.001
CRAT;CACP	4	0.7164	0.488(0.250−0.955)	0.036
LRIT2;LRIT2	4	0.8811	0.414(0.181−0.949)	0.037
PRR16;PRR16	4	0.9336	0.393(0.156−0.990)	0.048
CNGA2;CNGA2	4	0.9550	0.385(0.151−0.982)	0.046
ZG16;ZG16	4	1.0594	0.347(0.149−0.806)	0.014
TPI1;Triosephosphate−isomerase	3	1.1180	0.327(0.114−0.942)	0.038

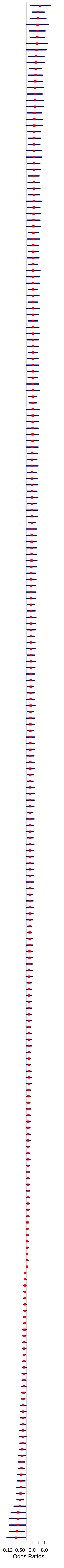

Supplement: Supplementary Figure 1 — The causality analysis result of the two-sample Mendelian randomization analysis shows 243 gene pairs for PCOS risk relationships. [file DataSheet1.pdf]
